# Association of Helicopter vs Ground Emergency Medical Transportation With 1-Year Mortality in Denmark

**DOI:** 10.1001/jamanetworkopen.2020.33318

**Published:** 2021-01-11

**Authors:** Karen Alstrup, Leif Rognås, Stephen Sollid, Søren Paaske Johnsen, Jan Brink Valentin, Jens Aage Kølsen Petersen

**Affiliations:** 1 Department of Research and Development, Prehospital Emergency Medical Services, Central Denmark Region, Aarhus, Denmark; 2 Department of Anaesthesiology, Aarhus University Hospital, Aarhus, Denmark; 3 Danish Air Ambulance, Denmark; 4 Department of Quality and Health Technology, Faculty of Health Sciences, University of Stavanger, Stavanger, Norway; 5 Norwegian Air Ambulance Foundation, Oslo, Norway; 6 Danish Center for Clinical Health Services Research, Department of Clinical Medicine, Aalborg University and Aalborg University Hospital, Aalborg, Denmark

## Abstract

**Importance:**

Appropriate use of helicopter emergency medical service (HEMS) is important in ensuring that patients with critical illness or injury receive adequate treatment.

**Objective:**

To investigate the association between use of HEMS compared with use of ground EMS (GEMS) and mortality overall and in a subgroup of patients with critical illness or injury.

**Design, Setting, and Participants:**

This register-based, nationwide cohort study used data retrieved from Danish registries from October 1, 2014, to April 30, 2018. Patients receiving GEMS originated from dispatched HEMS missions for which a helicopter was unavailable. For the primary analysis, patients from accepted HEMS missions and patients from missions in which HEMS was dispatched but unavailable were included. The secondary analysis included patients assigned a hospital *International Statistical Classification of Diseases and Related Health Problems, Tenth Revision *(*ICD-10*) diagnosis considered a critical illness or injury. These patients were selected via a consensus-based agreement among all authors by reviewing the Danish version of the World Health Organization’s *ICD-10* classification. Data were analyzed from March to June 2020.

**Exposures:**

Dispatch of HEMS vs GEMS unit (primary analysis) and treatment and transport by HEMS vs GEMS unit among patients with critical illness or injury (secondary analysis).

**Main Outcomes and Measures:**

One-year mortality was retrieved from the Danish Civil Registration System.

**Results:**

Among 10 618 patients (median [interquartile range] age, 60 [42-72] years; 6834 [64.4%] men) included in the primary analysis, 9480 patients (89.3%) received HEMS and 1138 patients (10.7%) received GEMS. Median (interquartile range) age was 60 (42-72) years, and 6834 patients (64.4%) were men. Adjusted cumulative 1-year mortality was 23.2% (95% CI, 22.4%-24.1%) among patients receiving HEMS vs 24.5% (95% CI, 21.9%-27.1%) among patients receiving GEMS. The difference in mortality risk for HEMS compared with GEMS was not statistically significant (hazard ratio, 0.94 [95% CI, 0.84-1.06]). Among 2260 patients with critical illness or injury receiving HEMS, compared with 315 patients with critical illness or injury receiving GEMS, adjusted cumulative 1-year mortality was 25.1% (95% CI, 23.5%-26.7%) vs 27.1% (95% CI, 22.0%-32.1%). The difference in mortality risk for HEMs compared with GEMs was not statistically significant (hazard ratio, 0.91 [95% CI, 0.73-1.14]).

**Conclusions and Relevance:**

This study found that 1 year after dispatch, the use of HEMS, compared with the use of GEMS, was not associated with a statistically significant difference in mortality overall or mortality among patients with critical illness or injury. Further research is needed to determine whether optimized dispatch systems may be associated with further improvements in survival among selected patients.

## Key Points

**Question:**

Is this use of a helicopter emergency medical service (HEMS) unit vs a ground EMS (GEMS) unit associated with different mortality rates among patients?

**Findings:**

In this cohort study of 10 618 patients in Denmark, the difference in adjusted cumulative 1-year mortality was not statistically significant among patients receiving HEMS compared with patients receiving GEMS.

**Meaning:**

This study found that the use of HEMS had no statistically significant association with 1-year mortality.

## Teaser

This cohort study assesses whether emergency medical transportation by helicopter vs by ground is associated with differences in mortality among patients in Denmark.

## Introduction

Helicopter emergency medical service (HEMS) is part of many prehospital health care systems. The main purpose of most European HEMS units is to bring advanced critical care to patients and provide rapid transportation to definitive care. Studies^1,2^ have found that a timely and goal-directed prehospital response is associated with improved morbidity and mortality in selected patient subgroups. However, there is inconsistent evidence in the literature for benefit associated with HEMS.^3,4^

We previously found^5^ that approximately two-thirds of patients attended to by physician-paramedic–staffed Danish HEMS have severe illness or injury and that the diagnostic groups most commonly seen consist of patients with time-critical conditions, such as cardiovascular emergencies, neurovascular emergencies, and severe trauma. Furthermore, we found^5^ that 14% of HEMS dispatches are rejected mainly owing to weather conditions below minimal HEMS operating requirements. Consequently, a group of patients triaged by the emergency medical dispatch centers (EMDCs) to be in need of HEMS was left to be attended solely by ground EMS (GEMS) units. The allocation of transportation modality based on weather conditions provided the opportunity to compare outcomes in patients with a different level of exposure (HEMS vs GEMS), mimicking what might have been achieved by a scientific randomized allocation of patients.

The primary aim of this study was to compare all-cause mortality among patients dispatched a HEMS unit with that among patients dispatched a GEMS unit in situations in which HEMS was unavailable, thus focusing on the current utilization of HEMS. The secondary aim was to compare mortality risk in the subgroup of patients with critical illness or injury treated and transported by a HEMS unit with that of patients with critical illness or injury treated and transported by a GEMS unit, thus focusing on an optimized use of HEMS.

## Method

### Study Design and Setting

This nationwide cohort quasi-experimental study was approved by the Danish National Board of Health and the Danish Data Protection Agency. An approval from the research ethics committee system and informed consent were not required for this observational study, as according to the Act on Research Ethics Review of Health Research Projects in Denmark, notification to the research ethics committee system of questionnaire surveys or medical database research projects (including informed consent) is required only if the project involves human biological material. The study complies with the Strengthening the Reporting of Observational Studies in Epidemiology (STROBE) reporting guideline.

The study was based on data from the national Danish helicopter database^6^ covering all HEMS dispatches in Denmark registered from October 1, 2014, to April 30, 2018. For dispatches in which HEMS was unavailable, the database holds operational data (ie, date and time and location of patient) of the dispatch. These missions constitute GEMS missions.

Denmark covers approximately 45 000 km^2^, with both urban and rural areas, including 70 smaller islands not connected by road to the mainland. The country is divided into 5 regions, with a total resident population of 5.8 million people.^7^ Health care in Denmark is a tax-supported service, free at the point of access. Each region has its own EMS and a varying number of district general hospitals capable of treating most common medical and surgical conditions, including less severe trauma. In addition, 4 university hospitals provide 24-hour neurosurgical treatment and thoracic surgical treatment, level 1 trauma care, thrombectomy for stroke, percutaneous coronary intervention, and pediatric intensive care. Dispatch of all EMS units is controlled from the 5 regional EMDCs. Emergency calls are handled by specially trained medical dispatchers using a national, criteria-based protocol when assessing the level of urgency and dispatching the proper response.^8,9^

Regional EMS centers are 3-tiered systems based on ambulances, ground-based prehospital critical care teams, and airborne prehospital critical care teams (ie, HEMS). Ambulances are staffed by emergency medical technicians or paramedics, whereas ground-based prehospital critical care teams consist of a consultant anesthesiologist (ie, a specialist covering anesthesia and intensive care, advanced pain management, and critical emergency medicine) and an emergency medical technician or a paramedic. The Danish EMS has been described in detail in studies from 2011 to 2019.^8,10,11,12^

The HEMS teams consist of a consultant anesthesiologist, a specially trained HEMS paramedic, and a pilot. Thus, Danish HEMS units offer a combination of treatment by an experienced and skilled HEMS crew, high-level decision-making and triage on scene, and fast transportation to definitive hospital care. During the study period, HEMS provided national coverage 24 hours a day and 7 days a week by operating 3 identical helicopters (EC 135 P3, Airbus Helicopters) equipped and certified for flying under visual and instrument flight rules using point-in-space navigation if necessary day and night, responding from 3 bases.

National HEMS dispatch criteria include primary missions based on emergency call (mainly time-critical conditions), secondary mission (interhospital transfers), and evacuation from the many smaller islands for logistic reasons. We previously described^5,6^ the HEMS dispatch protocol and the national Danish HEMS database in detail. In the eAppendix in the Supplement, HEMS dispatch and operational outcomes (ie, mission types) are specified, together with a table of HEMS minimal operating weather requirements (eTable 1 in the Supplement) and the cleaning of data.

### Selection of Patients

The selection process of patients for the primary and secondary analyses is displayed in the Figure. Patients included in the primary analysis were those from all accepted primary HEMS missions and patients from missions in which HEMS units were dispatched but unavailable (ie, the reference group). The exclusion criteria were telephone inquiries not leading to a HEMS mission, interhospital transfers (ie, secondary missions), patients with missing or incomplete Civil Registration System (CRS) numbers, missions with registration errors, and missions in which 2 or more CRS numbers were reported on the same HEMS dispatch.

**Figure.**
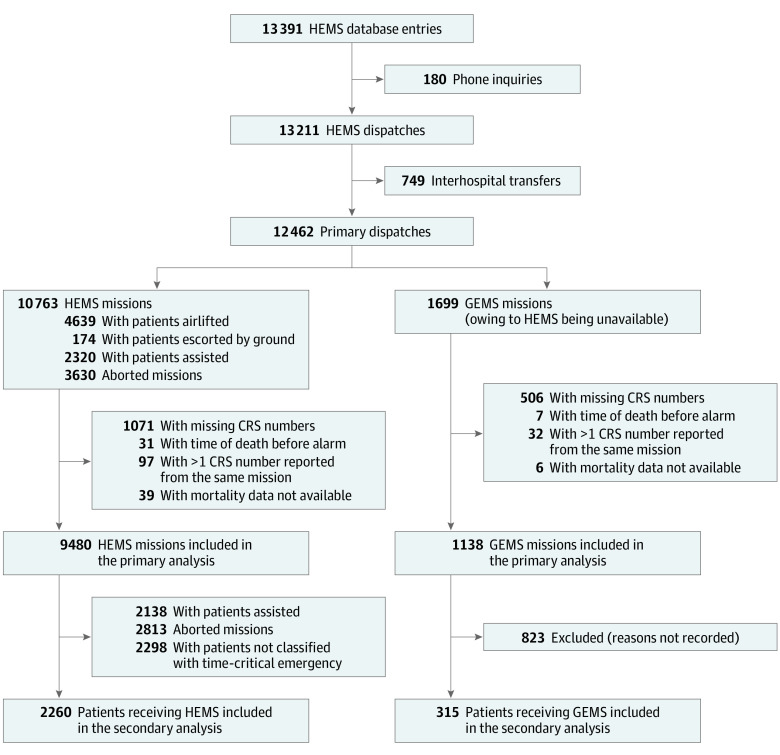
Flowchart of Patient Inclusion in the Primary and Secondary Analyses CRS indicates Civil Registration System; HEMS, helicopter emergency medical service; GEMS, ground EMS.

Patients included in the secondary analysis were those from accepted primary HEMS missions in which the patient was treated and transported to hospital by HEMS and assigned a hospital World Health Organization (WHO) *International Statistical Classification of Diseases and Related Health Problems, Tenth Revision *(*ICD-10*) diagnosis representing a time-critical condition (eg, cardiovascular or neurovascular emergency or a severe trauma) and patients who were treated and transported to hospital solely by GEMS owing to HEMS being unavailable and who were assigned a hospital WHO *ICD-10* diagnosis representing a time-critical condition (ie, reference group). Exclusion criteria were HEMS missions canceled en route (ie, aborted missions) and patients assisted by the HEMS team on scene but not escorted to hospital by HEMS (ie, patients receiving assistance).

All potentially relevant *ICD-10* diagnostic codes as classified by WHO were carefully reviewed. The selection of diagnoses was completed and agreed on in a consensus-based approach blinded for the exposure and the outcome variable. To evaluate diagnostic and prognostic similarity between patients receiving GEMS and those receiving HEMS, 12 subcategories of diagnoses were created (ie, multiple traumas; traumatic brain injury; thoracic and abdominal trauma and burns; trauma of the face or neck; trauma of extremities, amputation, and spinal injury; ST-segment–elevation myocardial infarction; non–ST-segment–elevation myocardial infarction; cardiac arrest; cardiac arrythmia; heart failure, pulmonary embolism, or aortic aneurysm; hemorrhagic stroke; and ischemic stroke or transitoric cerebral ischemia. A complete list of the selected *ICD-10* codes and the grouping of subcategories are displayed in the eTable 2 in the Supplement.

### Data Sources and Covariates

We retrieved operational data from the HEMS database and data on mortality from the Danish Civil Registration System containing updated vital status for all Danish citizens and noncitizens with permanent residency.^13,14^ From the Danish National Patient Register, we acquired data on first specific diagnosis according to the Danish version of *ICD-10*, comorbidity status divided into 4 groups,^15,16,17^ and hospital destination (Table 1 and Table 2). Individual-level linkage of data obtained from these databases was possible using the 10-digit personal CRS number assigned to each Danish citizen and foreign-born individuals with permanent residency in Denmark.

**Table 1.** Patient Demographic Characteristics and Comorbidity Groups

Characteristic	Patients, No. (%)		
	Receiving HEMS (n = 9480)	Receiving GEMS (n = 1138)	Total (N = 10 618)
Age, median (IQR), y	60 (41-72)	63 (45-74)	60 (42-72)
Men	6110 (64.5)	724 (63.6)	6834 (64.4)
Charlson comorbidity index group			
0	5757 (60.7)	624 (54.8)	6381 (60.1)
1-2	2282 (24.1)	317 (27.9)	2599 (24.5)
3-4	818 (8.6)	110 (9.7)	928 (8.7)
≥5	623 (6.6)	87 (7.6)	710 (6.7)

Abbreviations: GEMS, ground emergency medical service; HEMS, helicopter emergency medical service; IQR, interquartile range.

**Table 2.** Characteristics Among Patients With Critical Illness or Injury

Characteristic	Patients, No. (%)		
	Receiving HEMS (n = 2260)	Receiving GEMS (n = 315)	Total (N = 2575)
Age, median (IQR), y	62 (50-72)	66 (52-76)	63 (50-73)
Men	1620 (71.7)	216 (68.6)	1836 (71.3)
Charlson comorbidity index group			
0	1455 (64.4)	186 (59.0)	1641 (63.7)
1-2	528 (23.4)	78 (24.8)	606 (23.5)
3-4	192 (8.5)	30 (9.5)	222 (8.6)
≥5	95 (4.2)	21 (6.7)	116 (4.5)
Selected comorbidity^a^			
Acute myocardial infarction	140 (6.2)	16 (5.1)	156 (6.1)
Congestive heart failure	134 (5.9)	25 (7.9)	159 (6.2)
Peripheral vascular disease	145 (6.4)	29 (9.2)	174 (6.8)
Cerebral vascular disease	194 (8.6)	16 (5.1)	210 (8.2)
Chronic pulmonary disease	140 (6.2)	28 (8.9)	168 (6.5)
Diabetes	221 (9.8)	45 (14.3)	266 (10.3)
Any cancer	220 (9.8)	38 (12.1)	258 (10.0)
Diagnostic group			
Trauma	630 (27.9)	64 (20.3)	694 (27.0)
Cardiovascular emergency	1152 (51.0)	171 (54.3)	1323 (51.4)
Neurovascular emergency	478 (21.2)	80 (25.4)	558 (21.7)
Diagnostic subcategory			
Multiple traumas	160 (7.1)	25 (7.9)	185 (7.2)
Traumatic brain injury	141 (6.2)	16 (5.1)	157 (6.1)
Thoracic and abdominal trauma and burns	127 (5.6)	13 (4.1)	140 (5.4)
Trauma of the face or neck	40 (1.8)	<5^b^	43 (1.7)
Trauma of extremities, amputation, and spinal injury	162 (7.2)	7 (2.2)	169 (6.6)
ST-segment–elevation myocardial infarction	501 (22.2)	61 (19.4)	562 (21.8)
Non–ST-segment–elevation myocardial infarction	102 (4.5)	25 (7.9)	127 (4.9)
Cardiac arrest	386 (17.1)	57 (18.1)	443 (17.2)
Cardiac arrythmia	83 (3.7)	14 (4.4)	97 (3.8)
Heart failure, pulmonary embolism, or aortic aneurysm	80 (3.5)	14 (4.4)	94 (3.7)
Hemorrhagic stroke	179 (7.9)	33 (10.5)	212 (8.2)
Ischemic stroke or transitoric cerebral ischemia	299 (13.2)	47 (14.9)	346 (13.4)
Hospital destination			
University hospital	1861 (82.3)	153 (48.6)	2014 (78.2)
District general hospital	399 (17.7)	162 (51.4)	561 (21.8)
Secondary transfer to university hospital within 24 h	12 (3.0)	8 (4.9)	20 (3.6)
Secondary transfer to university hospital	221 (55.4)	78 (48.1)	299 (53.3)

Abbreviations: GEMS, ground emergency medical service; HEMS, helicopter emergency medical service; IQR, interquartile range.

^a^ Acute myocardial infarction, congestive heart failure, peripheral vascular disease, cerebral vascular disease, and chronic pulmonary disease were given a weight of 1 and diabetes and cancer were given a weight of 2 in the Charlson comorbidity index.

^b^ Owing to Danish law (patient discretion), it is not possible to report numbers less than 5.

### Exposures and Outcomes

In the primary analysis, patients were divided into 2 exposure groups by dispatch type: individuals dispatched a helicopter and individuals dispatched a GEMS unit because HEMS was unavailable (ie, the reference group). In the secondary analysis, patients were divided into 2 exposure groups by transportation modality: individuals treated and transported by the HEMS team and individuals treated and transported solely by GEMS because HEMS was unavailable (Figure).

The primary outcome was all-cause mortality at 1 year. Secondary outcomes included mortality at 1 day and 30 days of follow-up, transportation to a university hospital as the first receiving hospital, secondary transfer to a university hospital if first receiving hospital was a district general hospital, and numbers needed to fly (ie, the number of patients who need to be flown to prevent 1 additional death). Mortality at 1-day follow-up was defined as death on the same day as HEMS dispatch or the day after HEMS dispatch.

### Statistical Analysis

For the main analysis, we used the Kaplan-Meier failure curve to estimate cumulative mortality, including 95% CIs with administrative censoring at 1, 30, and 365 days of follow-up. Patients in the survival analysis were followed until death, emigration, or administrative censoring, whichever came first. In addition, we computed adjusted cumulative mortality using inverse probability of treatment weighting, adjusting for age, sex, and Charlson comorbidity index group in the primary analysis and age, sex, Charlson comorbidity index group, and diagnostic index group in the secondary analysis. Likewise, we computed inverse probability of treatment weighting–adjusted cumulative mortality differences and hazard ratios (HRs) with 95% CIs comparing patients receiving HEMS with those receiving GEMS using Cox proportional hazards regression. We conducted balance diagnostics of the inverse probability of treatment weighting analyses by assessing the standardized difference of potential confounders after weighting. A cutoff of 0.1 was applied. The numbers needed to fly value was calculated from the inverse of the difference in cumulative mortality between HEMS and GEMS at day 365.

All analyses were conducted using Stata statistical software version 15.1 (StataCorp), and significance was determined using 95% CIs in 2 ways: HRs were considered significant if 95% CIs did not cross 1, and cumulative mortality estimates were considered significant if the estimate for the HEMS group was not included in the GEMS group and vice versa. Data were analyzed from March 2020 to June 2020.

## Results

### Characteristics of Study Subjects

A total of 13 391 HEMS entries were registered in the database during the study period. Of these, 45 patients were lost to follow-up owing to emigration and hence no mortality data were available for them. In addition, 1571 patients’ CRS numbers were missing, and 602 of these numbers (38.3%) referred to the first year of service. Among 10 618 patients included in the primary analysis, median (interquartile range [IQR]) age was 60 (42-72) years and 6834 (64.4%) were men; 9480 patients (89.3%) received HEMS and 1138 patients (10.7%) received GEMS (Figure). The median (IQR) age was 60 (41-72) years among patients receiving HEMS and 63 (45-74) years among patients receiving GEMS. Among patients receiving HEMS, 6110 (64.5%) were men, and among those receiving GEMS, 724 (63.6%) were men. Overall, 6381 (60.1%) patients had no previously reported comorbidities (Table 1).

Among 2575 patients in the secondary analysis, 2260 patients (87.8%) were included in the HEMS group and 315 patients (12.2%) were included in the GEMS group. The median (IQR) age in the secondary analysis was 62 (50-72) years among patients receiving HEMS and 66 (52-76) years among patients receiving GEMS. Among patients receiving HEMS, 1620 (71.7%) were men, and among patients receiving GEMS, 216 (68.6%) were men. Among patients with critical illness or injury, 1641 patients (63.7%) had no previously reported comorbidities (Table 2). The distribution of diagnostic subcategories was similar in the 2 groups.

### Mortality

Adjusted cumulative mortality for patients receiving HEMS compared with patients receiving GEMS was 14.4% (95% CI, 13.6%-15.1%) vs 15.5% (95% CI, 13.3%-17.7%) at day 1, 19.1% (95% CI, 18.2%-19.9%) vs 20.0% (95% CI, 17.7%-22.3%) at day 30, and 23.2% (95% CI, 22.4%-24.1%) vs 24.5% (95% CI, 21.9%-27.1%) at day 365. The difference in mortality risk for HEMS compared with GEMS was not statistically significant (HR, 0.94 [95% CI, 0.84-1.06]) (Table 3).

**Table 3.** Mortality Among Patients Receiving HEMS or GEMS

Outcome	Mortality, % (95% CI)		
	Receiving HEMS (n = 9480)	Receiving GEMS (n = 1138)	Difference
Crude cumulative mortality			
Day 1	14.3 (13.6 to 15.0)	16.2 (14.2 to 18.4)	1.7 (−0.6 to 4.1)
Day 30	18.9 (18.2 to 19.7)	21.0 (18.8 to 23.5)	1.9 (−0.6 to 4.4)
Day 365	23.0 (22.2 to 23.9)	26.0 (23.6 to 28.7)	2.8 (0.0 to 5.6)
Adjusted cumulative mortality			
Day 1	14.4 (13.6 to 15.1)	15.5 (13.3 to 17.7)	1.1 (−1.2 to 3.4)
Day 30	19.1 (18.2 to 19.9)	20.0 (17.7 to 22.3)	0.8 (−1.6 to 3.3)
Day 365	23.2 (22.4 to 24.1)	24.5 (21.9 to 27.1)	1.3 (−1.4 to 4.0)
Adjusted HR (95% CI)	0.94 (0.84 to 1.06)	1 [Reference]	NA

Abbreviations: GEMS, ground emergency medical service; HEMS, helicopter emergency medical service; HR, hazard ratio; NA, not applicable.

In the subgroup of patients with critical illness or injury, cumulative mortality among patients receiving HEMS compared with patients receiving GEMS was 11.3% (95% CI, 10.1%-12.6%) vs 13.8% (95% CI, 9.6%-18.0%) at day 1, 21.8% (95% CI, 20.3%-23.4%) vs 22.6% (95% CI, 17.8%-27.4%) at day 30, and 25.1% (95% CI, 23.5%-26.7%) vs 27.1% (95% CI, 22.0%-32.1%) at day 365, (Table 4). The difference in mortality risk for HEMS compared with GEMS was not statistically significant (HR, 0.91 [95% CI, 0.73-1.14]). Kaplan-Meier mortality estimates are presented in eFigure 1 and eFigure 2 in the Supplement.

**Table 4.** Mortality Among Patients With Critical Illness or Injury

Outcome	Mortality, % (95% CI)		
	Receiving HEMS (n = 2260)	Receiving GEMS (n = 315)	Difference
Crude cumulative mortality			
Day 1	11.2 (10.0 to 12.6)	14.9 (11.4 to 19.4)	3.7 (−0.7 to 8.3)
Day 30	21.6 (20.0 to 23.4)	25.1 (20.7 to 30.3)	3.5 (−1.6 to 8.8)
Day 365	24.8 (23.1 to 26.7)	30.8 (26.0 to 36.2)	6.0 (0.8 to 11.5)
Adjusted cumulative mortality			
Day 1	11.3 (10.1 to 12.6)	13.8 (9.6 to 18.0)	2.4 (−1.9 to 6.8)
Day 30	21.8 (20.3 to 23.4)	22.6 (17.8 to 27.4)	0.8 (−4.2 to 5.8)
Day 365	25.1 (23.5 to 26.7)	27.1 (22.0 to 32.1)	2.1 (−3.1 to 7.2)
HR (95% CI)	0.91 (0.73 to 1.14)	1 [Reference]	NA

Abbreviations: GEMS, ground emergency medical service; HEMS, helicopter emergency medical service; HR, hazard ratio; NA, not applicable.

Presuming that the difference in cumulative mortality was associated solely with the difference in transportation modality, the number needed to fly for HEMS to be associated with an increase of 1 additional life saved was 47. All standardized differences of the adjustment variables were less than 0.1 after inverse probability of treatment weighing.

## Discussion

This cohort study of the Danish nationwide HEMS did not find any statistically significant difference in adjusted mortality in patients dispatched a HEMS unit compared with patients dispatched a GEMS unit. In the subgroup of patients with critical illness or injury, there was a lower mortality among patients receiving HEMS compared with patients receiving GEMS, although this difference was not statistically significant. Further research is needed to determine whether proper HEMS dispatch may be of importance for improved patient outcome in selected patients.

Although observational in nature, the study design is a major strength of this study, simulating a randomized design by allocation of patients into the exposure groups. We took advantage of the fact that some patients were treated and transported solely by GEMS due to the unavailability of HEMS. Decisions to reject HEMS missions were based on factors related to flight operations only, mainly poor weather conditions. To our knowledge, this study is the first of its kind to apply this approach simulating a randomized study design.

In addition to the instances in which the HEMS team escorted the patient to hospital, the primary analysis also included aborted missions and patients who were assisted. These instances accounted for more than half of the HEMS missions, and they may partly reflect HEMS overtriage, as discussed in our 2019 study.^5^

Among patients receiving HEMS, compared with patients receiving GEMS, a 2020 study by Beaumont et al^18^ found a 15% nonsignificant decrease in mortality risk and Stewart et al^19^ found a 33% decrease in mortality risk. A positive association between HEMS and survival was also found in 2 other propensity-matched studies.^20,21^ Tsuchiya et al^20^ further found a number needed to fly ranging from 15 to 46 using different analysis strategies. Study settings and target populations were not identical to those of our study.

More than half of the patients in the GEMS group in our study were transported to a district general hospital, compared with approximately 18% in the HEMS group. Nearly half of these patients transported by GEMS were secondarily transported to a university hospital. This seems to underline the severity of their emergency conditions and the need of high-level specialized care and highlights the fact that HEMS in Denmark adds advanced clinical care, critical decision-making, and triage and also facilitates rapid transport to university hospitals. In most instances with no physician on scene, the ambulance crew are instructed to bring the patient to the nearest hospital, while triage made by an on-scene physician allows for direct patient transfer to specialized care (ie, university hospitals), thus bypassing smaller hospitals.^22^ The association this may have with estimated mortality in this study is unknown and requires further examination.

A 2011 study^1^ and a 2010 study^23^ found that health care system delay, defined as time from first contact with the health care system (via the Denmark emergency number [1-1-2] or general practitioners) to treatment (ie, reperfusion therapy), was associated with increased mortality and morbidity in patients with ST-segment–elevation myocardial infarction. Furthermore, a 2016 study^2^ and a 2003 study^24^ found a positive association between timely access to definitive care after traumatic injury and timely treatment (ie, thrombectomy) and outcome in patients who experienced stroke with a large vessel occlusion. In a 2019 Norwegian study^25^ of patient outcomes when HEMS was unavailable, lack of rapid transportation was the primary factor associated with lost life years. In summary, these studies support the importance of optimal prehospital treatment and triage as critical components in caring for patients in critical condition.

The wide 95% CIs in our study, which include the value of 1 revealing nonsignificant results, could be associated with the low number of patients in the GEMS group. As a result, our study results should be interpreted with caution. We believe that our findings may have a clinical impact. We suggest that efforts put into improved education of staff making triage decisions in EMDCs or in the field, together with the creation of validated dispatching tools, may be important in selecting the patients who are most likely to benefit from HEMS dispatch.

### Limitations

This study has several limitations, including the substantial proportion of missing CRS numbers, especially among patients receiving GEMS. The lack of follow-up for these patients was potentially associated with a lack of study robustness owing to possible selection bias. It appeared from our data that almost 40% of the missing CRS numbers referred to the first year of service, a period when CRS numbers were registered manually in the EMDC system after being handed over to the registration staff by a telephone call from the crew on scene. This could have been associated with registration errors. Although lacking in certainty, we consider the missing CRS numbers to be missing at random.

To our knowledge, there is no consensus regarding which groups of patients with emergency conditions may benefit from immediate specialized care. The patient groups selected for this study as being time critical may thus not represent the only patients who were truly time critical. Regarding the selection of trauma cases, an injury severity score greater than 15 used as an indicator of severe trauma could have been preferable. However, injury severity scores were not available in our system.

Additionally, seasonal variation in disease patterns and the association of certain weather conditions with the occurrence of critical illness and injuries are not well studied. We sought to reduce this potential selection bias related to prognostic differences between the exposure groups by adjusting for diagnoses in the secondary analyses.

## Conclusions

This cohort study’s findings suggest that 1 year after dispatch, the use of HEMS was not associated with a statistically significant difference in overall mortality or in mortality among patients with critical illness or injury compared with the use of GEMS. However, our results found that among these patients with critical illness or injury, mortality was lower in those receiving HEMS compared with those receiving GEMS, although the difference was not significant. This reduction in selected patients should be investigated in future studies.

## Notes

Supplement. **eAppendix.** Supplementary Methods **eTable 1.** HEMS Operating Weather Minima **eTable 2.** List of Included *ICD-10* Diagnoses **eFigure 1.** Kaplan-Meier Cumulative Mortality Estimates Among Patients Receiving HEMS or GEMS **eFigure 2.** Kaplan-Meier Cumulative Mortality Estimates Among Patients With Critical Illness or Injury Receiving HEMS or GEMS
