# Supplementary material for: Association of Helicopter vs Ground Emergency Medical Transportation With 1-Year Mortality in Denmark
Source: JAMA Netw Open. 2021 Jan 11;4(1):e2033318. doi: 10.1001/jamanetworkopen.2020.33318 (PMC7801934; doi:10.1001/jamanetworkopen.2020.33318)
Supplement: Supplement. — eAppendix. Supplementary Methods eTable 1. HEMS Operating Weather Minima eTable 2. List of Included ICD-10 Diagnoses eFigure 1. Kaplan-Meier Cumulative Mortality Estimates Among Patients Receiving HEMS or GEMS eFigure 2. Kaplan-Meier Cumulative Mortality Estimates Among Patients With Critical Illness or Injury Receiving HEMS or GEMS [file jamanetwopen-e2033318-s001.pdf]

Supplemental Online Content

Alstrup K, Rognås L, Sollid S, Johnsen SP, Valentin JB, Petersen JAK. Association of helicopter vs ground emergency medical transportation with 1-year mortality in Denmark. *JAMA Netw Open*. 2021;4(1):e2033318. doi:10.1001/jamanetworkopen.2020.33318

eAppendix. Supplementary Methods
eTable 1. HEMS Operating Weather Minima
eTable 2. List of Included *ICD-10* Diagnoses
eFigure 1. Kaplan-Meier Cumulative Mortality Estimates Among Patients Receiving HEMS or GEMS
eFigure 2. Kaplan-Meier Cumulative Mortality Estimates Among Patients With Critical Illness or Injury Receiving HEMS or GEMS

This supplemental material has been provided by the authors to give readers additional information about their work.

© 2021 Alstrup K et al. *JAMA Network Open.*

eAppendix. Supplementary Methods

Main HEMS dispatch criteria, HEMS operational outcomes, reason for aborted and rejected missions, the judgement of an unavailable HEMS mission, and data cleaning.

In the Danish setting, the defined criteria for dispatch are a suspected time-critical condition (stroke, acute myocardial infarction, cardiac arrest, and severe trauma). HEMS may be dispatched immediately based on a 1-1-2 call, or on crew request from the scene by first attending ground unit based on the above-mentioned dispatching criteria. A HEMS dispatch may result in four different operational outcomes: i) the patient being escorted to hospital by the HEMS team, either by air (air lifted patients) or land (ground escorted patients), ii) the HEMS team attending the patient and assisting the ground crew but do not escort the patient to hospital (assisted patients), iii) HEMS being cancelled en-route before reaching the patient; most often based on information from the land crew first on scene (aborted missions), and iv) the mission being rejected due to the unavailability of HEMS (rejected missions). The small subset of patients among the HEMS missions that were ground escorted to hospital by a HEMS physician in an ambulance instead of being air lifted (n=174), refer to the HEMS missions as HEMS personnel were involved in the transportation.
It is the responsibility of the HEMS pilot to assess whether a HEMS mission may be completed or not. Reasons for rejecting a mission are listed below, and it appears that adverse weather conditions are the primary reason for a rejection. The eTable1 "HEMS Operating weather minima" (below) summarizes the standards that have to be met for completing a mission. The main reason for allocating a patient into intervention (HEMS) or control (GEMS) is thus based on HEMS operating weather minima assessed by the pilot independent of the condition of the patient.
As opposed to the organisation of other HEMS [1,2], the Danish HEMS team do not have the possibly to reach the patients by car in case of unavailable HEMS. Consequently, the missions rejected by HEMS are dealt with solely by ground units (GEMS missions).
The reason for aborted and rejected missions are presented in the two boxes below together with the cleaning of data. Geographical challenges making landing in reasonable vicinity of the patient impossible, adverse weather and technical issues were considered flight operational conditions not related to the clinical status of the patient. These missions were therefore reclassified to be included in the rejected mission group. In cases where the reason for rejecting the mission were categorised as "No need for HEMS", this was based on a patient assessment from the first arriving team and these missions were reclassified as an aborted mission.

**Reasons for rejected missions:**		**Reasons for aborted missions:**
Landing impossible 17	←	Landing impossible 4
Weather 1,242	←	Weather 225
Concurrency 16		Redirected 65
Technical issues 14	←	Technic issues 6
Other 93		Other 66
		HEMS stood down by RRV 918
		HEMS stood down by EMDC 1,219
		HEMS stood down by ambulance 952
No need for HEMS 394	→	
Duty-time regulations 9		
No time-gain from using HEMS 68		
Missing data 5		Missing data 11
**Total after reclassifying of missions N = 1,699**		**Total after reclassifying of missions N = 3,630**

© 2021 Alstrup K et al. *JAMA Network Open.*

**eTable 1** HEMS Operating Weather Minima	
**DAY**	
Ceiling	*Visibility*
500 feet and above	1500 meters
499-400 feet	2000 meters
399-300 feet	3000 meters
**NIGTH**	
Cloud base	*Visibility*
1200 feet	3000 meters

© 2021 Alstrup K et al. *JAMA Network Open.*

eTable 2. List of Included *ICD-10* Diagnoses

Category	ICD-10 codes
**Cardio-vascular emergencies**	
ST-elevation myocardial infarction	DI210B; DI211B; DI213; DI219
Non-ST-elevation myocardial infarction	DI210A; DI211A; DI214; DI248
Cardiac arythmia	DI442; DI495; DI470; DI472; DI490
Cardiac arrest	DI460; DI461; DI469
Heart failure	DI501A; DI501B; DI301; DI312; DI319A
Pulmonary embolism	DI260; DI269
Aortic aneurism	DI710; DI711; DI712; DI713; DI714; DI715; DI716; DI718
Stroke - ischemic	DI649; DI670; DI676; DI678A; DI630; DI631; DI632; DI633; DI634; DI635; DI636; DI638; DI639
Stroke - hemorrhagic	DI600; DI601; DI602; DI603; DI604; DI605; DI606; DI607; DI608; DI609; DI610; DI611; DI612; DI613; DI614; DI615; DI616; DI618; DI619; DI620; DI621; DI629
**Neurovascular emergencies**	
Transient cerebral ischemia	DG450; DG452; DG453; DG454; DG458; DG459A
**Trauma**	
Traumatic brain injury	DS061; DS062; DS063; DS064; DS065; DS066; DS067; DS068; DS069; DS097
Trauma - face and neck	DS020; DS021; DS027; DS028; DS029; DS07; DS100; DS15; DS17
Trauma - thorax	DS222; DS223; DS224; DS225; DS228; DS229; DS250; DS251; DS252; DS253; DS254; DS255; DS257; DS258; DS259; DS260; DS268; DS269; DS270; DS271; DS272; DS273; DS274; DS275; DS276; DS277; DS278; DS279; DS280; DS290; DS297; DS298; DS299
Trauma - abdomen	DS300; DS301; DS321; DS322; DS323; DS324; DS325; DS327B; DS328B; DS328C; DS334; DS350; DS351; DS352; DS353; DS354; DS355; DS357; DS358; DS359; DS360; DS361; DS362; DS363; DS364; DS365; DS366; DS367; DS368; DS369; DS37; DS381; DS397
Traumatic amputation	DS080; DS081; DS088; DS089; DS480; DS481; DS489; DS580; DS581; DS589; DS680; DS681; DS682; DS683; DS684; DS688; DS689; DS780; DS781; DS789; DS880; DS881; DS889; DS980; DS981; DS982; DS983; DS984; DT05
Trauma - upper and lower extremities	DS770; DS771; DS772; DS870; DS878; DT022; DT023; DT024; DT025; DT026; DT029
Traumatic spinal injury	DS12; DS140; DS141; DS22; DS240; DS241; DS320; DS321; DS322; DS324; DS325; DS327A; DS328A
Burns	DT203; DT213; DT223; DT233; DT243; DT253
Muliple injuries	DT020; DT021; DT027; DT040; DT041; DT042; DT043; DT044; DT047; DT048; DT049; DT079

© 2021 Alstrup K et al. *JAMA Network Open.*

eFigure 1. Kaplan-Meier Cumulative Mortality Estimates Among Patients Receiving HEMS or GEMS

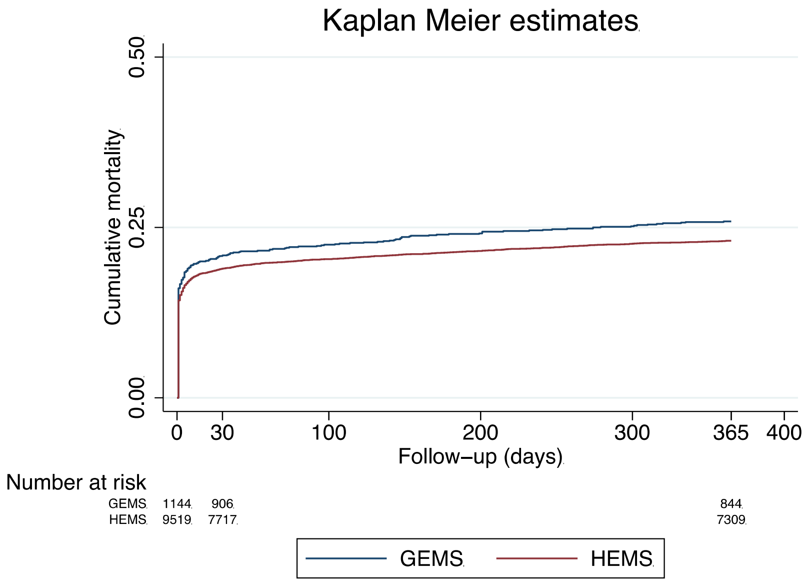

© 2021 Alstrup K et al. *JAMA Network Open.*

eFigure 2. Kaplan-Meier Cumulative Mortality Estimates Among Patients With Critical Illness or Injury Receiving HEMS or GEMS

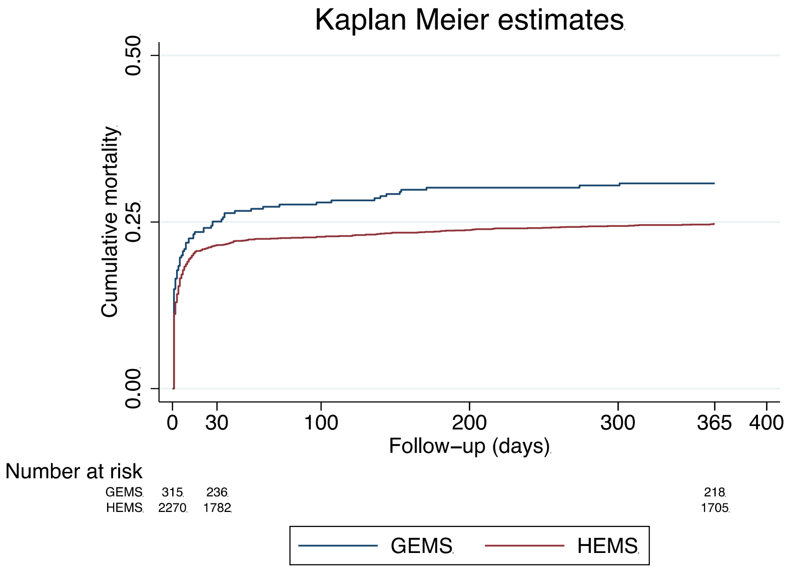

1. Saviluoto A, Björkman J, Olkinuora A, et al. The first seven years of nationally organized helicopter emergency medical services in Finland - the data from quality registry. *Scandinavian journal of trauma, resuscitation and emergency medicine.* 2020;28(1):46.
2. Osteras O, Brattebo G, Heltne JK. Helicopter-based emergency medical services for a sparsely populated region: A study of 42,500 dispatches. *Acta anaesthesiologica Scandinavica.* 2016;60(5):659-667.

© 2021 Alstrup K et al. *JAMA Network Open.*
